# Spatio-temporal patterning of different connexins in developing and postnatal human kidneys and in nephrotic syndrome of the Finnish type (CNF)

**DOI:** 10.1038/s41598-020-65777-5

**Published:** 2020-05-29

**Authors:** Ivona Kosovic, Natalija Filipovic, Benjamin Benzon, Katarina Vukojevic, Marijan Saraga, Merica Glavina Durdov, Ivana Bocina, Mirna Saraga-Babic

**Affiliations:** 1 Department of Anatomy, Histology and Embryology, School of Medicine, University of Split, Split, Croatia; 2 Department of Histology and Embryology, School of Medicine, University of Mostar, Mostar, Bosnia and Herzegovina; 3 Department of Paediatrics, University Hospital in Split, School of Medicine, University of Split, Split, Croatia; 4 Department of Pathology, University Hospital in Split, School of Medicine, University of Split, Split, Croatia; 5 Department of Biology, Faculty of Science, University of Split, Split, Croatia

**Keywords:** Developmental biology, Biomarkers, Nephrology

## Abstract

Connexins (Cxs) are membrane-spanning proteins which enable flow of information important for kidney homeostasis. Changes in their spatiotemporal patterning characterize blood vessel abnormalities and chronic kidney diseases (CKD). We analysed spatiotemporal expression of Cx37, Cx40, Cx43 and Cx45 in nephron and glomerular cells of developing, postnatal kidneys, and nephrotic syndrome of the Finnish type (CNF) by using immunohistochemistry, statistical methods and electron microscopy. During kidney development, strong Cx45 expression in proximal tubules and decreasing expression in glomeruli was observed. In developing distal nephron, Cx37 and Cx40 showed moderate-to-strong expression, while weak Cx43 expression gradually increased. Cx45/Cx40 co-localized in mesangial and granular cells. Cx43 /Cx45 co-localized in podocytes, mesangial and parietal epithelial cells, and with podocyte markers (synaptopodin, nephrin). Different Cxs co-expressed with endothelial (CD31) and VSMC (α –SMA) markers in vascular walls. Peak signalling of Cx37, Cx43 and Cx40 accompanied kidney nephrogenesis, while strongest Cx45 signalling paralleled nephron maturation. Spatiotemporal Cxs patterning indicate participation of Cx45 in differentiation of proximal tubules, and Cx43, Cx37 and Cx40 in distal tubules differentiation. CNF characterized disorganized Cx45 expression in proximal tubules, increased Cx43 expression in distal tubules and overall elevation of Cx40 and Cx37, while Cx40 co-localized with increased number of interstitial myofibroblasts.

## Introduction

During normal human kidney development, the uretheric bud induces the nearby metanephric mesenchyme to transform into metanephric cup cells, which than undergo through several developmental stages, including the renal vesicle stage, S-body and capillary loop stages, subsequently leading to mature nephron and glomeruli formation. Nephrogenesis is characterized by the mesenchymal to epithelial transition (MET) of future nephron cells, while podocytes also display a reverse process of epithelial to mesenchymal transition (EMT) at later developmental stages^1^. Podocytes are the key cells in formation of glomerular basement membrane (GBM), which increasingly expresses nephrin^2^ and synaptopodyn, cytoskeletal proteins critical for formation of the podocyte foot processes^3^. During normal kidney development, α –smooth actin (α –SMA) is increasingly expressed in smooth muscle cells in the walls of blood vessels (VSMC), pericytes and podocytes^1^, while the endothelium of blood vessels strongly expresses CD31 in the nephrogenic zone of the mouse^4^ and human developing kidneys^1^. Experimental animal models associate CD31 expression with development of atherosclerosis^5^. In chronic kidney disease (CKD), stronger expression of α –SMA actin is associated with the increased number of myofibroblasts in the interstitium of sclerotic kidneys, and with revascularization and regeneration of the affected kidneys^4,6^. CKD caused by the nephrotic syndrome of the Finnish type CNF) also shows changes in cytoskeletal filaments^1^. Recent studies indicate that in advanced stages of kidney disease, the amount of synaptopodyn in the urine can predict the extent of glomerular damage^7^.

Intercellular signaling via connexins (Cxs) has a major role both during normal kidney development, as well as in postnatal period and in progression of pathological changes. Cxs are membrane-spanning proteins, which by forming cell-to-cell channels and cell-to-extracellular space hemi-channels^8^ control cell proliferation and cell death^9,10^ as well as flow of information important for functional homeostasis within tissues and organs^11^. Cxs are widely spread in different organs, particularly in the vasculature, where they regulate vasomotion^12^. Therefore, alterations in normal Cx patterning are primarily observed in structural and functional changes of blood vessels^13^ and in severe vascular abnormalities^14^. Thus, acceleration of atherosclerosis was associated with Cx37 null mice^15^. While deletion of Cx45 was lethal in mouse development because of interrupted vessels formation^16,17^, its elevation led to development of hypertension^18^. Renal vasculature and the juxtaglomerular apparatus (JGA) have been shown to express Cx43, Cx40, Cx45 and Cx37, thus pointing to the role of those Cxs in several regulatory mechanisms within kidneys, including blood pressure control^19,20^.

Expression of different Cxs in tubular nephron cells and glomerular cells has been mostly analysed in rat and mice postnatal kidneys, but rarely during kidney development. Expression of various subtypes of Cxs was found along the kidney nephrons^20,21^, but clear morphological evidence for the presence of gap junctions has been reported only for their proximal tubules^20^. Expression of Cx37 was found in distal nephron^20^, in ascending limb and distal convoluted kidney tubules, while proximal tubules characterized its weaker expression^22^. Cx45 expression was observed in developing distal tubules of mice kidneys as well^23^.

Kidney glomeruli are structures which harbor several cells population and glomerular blood vessels, which display expression of different Cxs. Studies on Cxs expression in glomerular endothelial cells showed contradicting results^24–26^. Investigations on experimental animals reported that mesangial cells and podocytes can express Cx43^26–30^ and possibly Cx45^31^. In addition, mesangial cells expressed Cx37 and Cx40^24^.

In developing rat kidneys, Cx45 was detected in glomeruli and distal tubules^23^, while Cx37, Cx45 and Cx46 characterized early stages of rat development^32^. In human mid-term fetuses, Cx43 expression was reported in tubules of the renal cortex^33^, while recent study on human fetuses reported Cx36 expression in proximal tubules and collecting ducts, and weakly in Henle’s loop and distal tubules, while Cx 43 expression characterized proximal tubules and glomeruli^34^. During normal EMT of human kidney stem cell cultures Cx43 expression gradually decreased^35^.

Different types of CKD share common pathophysiological mechanism, which includes inflammation and pathological accumulation of extracellular matrix leading to scar formation^36^. Decrease of Cx37 was observed in CKD kidneys, while increase in Cx43 characterised inflammatory cells, tubular, interstitial cells and in the endothelium of capillaries in hypertensive nephropathy^37^. However, data on the role of Cx43 in kidney pathology remained contradicting^21^: while Cx43 upregulation in podocytes characterized rat experimental glomerulonephritis and type 2 diabetes, its downregulation was seen in overt diabetic nephropathy. Therefore, Cx43 could be considered as marker for podocyte injury^28,29^.

Despite numerous investigations on the role of connexins in kidney homeostasis, there are still evident discrepancies about kidney regions specifically expressing different Cx isoforms. There is only one experimental study comparing expression of Cx in foetal and adult kidney tissue^38^, while data on the Cxs expression outside the vascular wall are still largely unknown. This specially refers small number of studies on human developing kidneys, which cover relatively short developmental period^33,34^. The aim of the present study was to describe changes in spatiotemporal expression of Cxs in developing and postnatal human kidneys, as well as their precise localization within different kidney cells by using co-expression studies with synaptopodyn, nephrin, CD31 and α –SMA. In addition, we compared intensity of signal between healthy kidneys and nephrotic syndrome of the Finnish type (CNF) in the light of possible faulty intercellular signalling associated with end- stage renal disease.

## Materials and methods

### Human tissue processing

In our study, 11 human embryonic and foetal tissues were acquired from the Department of Gynaecology and Obstetrics after spontaneous abortions, or after tubal pregnancies from the Department of Pathology. Tissues were processed with permission of the Ethical and Drug Committee of the University Hospital in Split in accordance with Helsinki Declaration. The age of conceptuses was evaluated between 8^th^ and 38^th^ developmental week from menstrual data and corresponding to the external measurements (crown–rump length) and the Carnegie staging system. All tissues were without sings of macerations and morphologically regular. In our research we also included postnatal tissue taken during autopsy of healthy 1,5- year old boy and nephrotic kidney tissue from 3 nephrectomised CNF patients (homozygous missense mutation c.1096 A > C; pSer366Arg in NPHS1 gene was detected in all three patients). Informed parental consent was obtained for the research purposes^2^. Tissues were furtherly processed and glass slides were prepared as previously described^39^. Tissue was fixed in 4% paraformaldehyde in phosphate buffer saline (PBS), dehydrated in ethanol dilutions, paraffin-embedded and serially cut as 5 µm thick sections. Appropriate tissue preservation was confirmed by Haematoxylin and Eosin staining of every 10^th^ section. Tissue was analyzed using Olympus BX51 light microscope (Olympus, Tokyo, Japan).

### Immunohistochemistry and immunofluorescence staining

Tissue sections were deparaffinized and then rehydrated in ethanol following standard protocol^40^ and treated with sodium citrate buffer. After washing with PBS, blocking buffer (Protein Block, Abcam, UK) was administered on the tissue covered area. Primary antibodies were applied (Table 1) overnight and rinsed in PBS. Suitable secondary antibodies were applied (Table 1) and incubated in humidity chamber for one hour. Considering the double staining with lectins, after the mentioned procedure of administering primary antibodies, sections were incubated with FITC-conjugated lectins (Dolichos Biforus Agglutinin - DBA or Lotus Tetragonolobus Lectin - LTL, Table 1) in dark. They were washed in PBS following the 2 hr incubation period at room temperature^41^. Slides were afterwards washed in PBS and treated with DAPI nuclear staining. Control for specificity was excluding primary antibody from the staining procedure. For imaging, Olympus fluorescence microscope (BX61; Tokyo, Japan) with a digital camera (DP71) was used. Images were captured by using the Olympus CellA software and assembled in the plates by Adobe Photoshop. For Fig. 5 exclusively, we used DS-Ri2 digital camera for imaging, and NIS-Elements F software for image capture.

**Table 1:** Primary and secondary antibodies used in the study.

	Antibodies	Host	Dilution	Source
Primary	Anti-Cx37/GJA4 ab181701	Rabbit	1:500	Abcam (Cambridge, UK)
	Anti-Cx40/GJA5 ab213688	Rabbit	1:100	Abcam (Cambridge, UK)
	Anti-Cx43&GJA1 ab87645	Goat	1:200	Abcam (Cambridge, UK)
	Anti-Cx45/GJA7 ab135474	Rabbit	1:100	Abcam (Cambridge, UK)
	Purified Mouse Anti-Rat CD31 Clone TLD-3A12	Mouse	1:100	BD Pharmingen™ (CA, USA)
	Smooth Muscle Actin (**M0851**)	Mouse	1:200	Dako (Denmark)
	Nephrin Ab (G-20) sc-32530	Goat	1:200	Santa Cruz Biotechnology, Inc., (Santa Cruz, CA, USA)
	Anti-Synaptopodin antibody ab117702	Rabbit	1:300	Abcam (Cambridge, UK)
Secondary	Anti-Goat IgG, Alexa Fluor® 488, ab150129	Donkey	1:400	Abcam (Cambridge, UK)
	Anti-Rabbit IgG, Alexa Fluor® 488, 711-545-152	Donkey	1:400	Jackson Immuno Research Laboratories, Inc., Baltimore, PA, USA
	Anti-Mouse IgG, Alexa Fluor® 488 ab150105	Donkey	1:400	Abcam (Cambridge, UK)
Lectins	Fluorescein labeled Dolichos Biflorus Agglutinin (DBA) FL-1031	—	1:300	Vector Laboratories Ltd., Peterborough, UK
	Fluorescein labeled Lotus Tetragonolobus Lectin (LTL) FL-1321	—	1:300	Vector Laboratories Ltd., Peterborough, UK

### Semi thin and ultra-thin sections and electron microscopy

In preparation for electron microscopy we used tissue samples of 10^th^ and 22^nd^ week-old human kidneys, 1.5 years healthy and 3-year CNF kidney tissue. After the 24- hour fixation in 4% paraformaldehyde, specimens were post-fixed in 1% osmium tetroxide for an hour, dehydrated in courses of ethanol and embedded in LX 112 resin. Toluidine blue was used for staining of previously cut semi thin sections (1 µm). Semi thin sections were additionally cut into ultrathin sections (0.05 µm thick) which were examined by transmission electron microscope (Zeiss 902 A, Germany) after being treated with uranyl acetate and lead citrate staining^2^.

### Semi-quantification

The staining intensity of chosen antibodies was semi-quantitatively assessed by four categories: with (−) indicating the absence of any reactivity; (+) as a mild reactivity; (++) as moderate reactivity; and (+++) as strong reactivity (Table 2). Three researchers semi-quantitatively analyzed the staining intensity separately by image analysis software ImageJ (National Institutes of Health, Bethesda, MD, USA)^1^.

**Table 2:** Expression of different Cxs, synaptopodin, CD31 and α –SMA in the developing kidneys, postnatal healthy and CNF kidneys.

WEEKS/YEARS	PART OF NEPHRON	Cx43	Cx45	Cx40	Cx37	Syn	CD31	α –SMA
8–10 weeks	metanephric cup	+	+++	++	++/+++	−	++	++
	immature glomerulus	+	+++	++/+++	+	−	++	++
	collecting tubule	+	++/+++	+++	+++	−	−	−
21–22 weeks	glomerulus	+	++	++	++	++	+++	+++
	proximal convoluted tubule	+	+++	+++	+	+	−	−
	distal tubule	+	++	++	++	+	−	−
38 weeks	glomerulus	+	++	++	++	+++	+++	+++
	proximal convoluted tubule	+	+++	++	+	+	−	−
	distal tubule	++	++	++	++	+	−	−
1,5 year	glomerulus	+/+ +	+++	+	++	+++	+++	+++
	proximal convoluted tubule	++	+++	++	+	+	−	−
	distal tubule	++	++	+++	++	+	−	−
CNF - cca 1,5 year	glomerulus	++	+++	++	+++	++	+/++	++
	proximal convoluted tubule	++	+++	++	+/−	+/−	−	−
	distal tubule	+++	++	+++	+++	+/−	−	−

(−) absence of any reactivity; (+) mild reactivity; (++) moderate reactivity; and (+++) strong reactivity.

### Statistical analysis

Florescence intensity histograms were acquired for red and green fluorescence channels in ImageJ software (NIH, Bethesda, MD, USA). Pixels that had fluorescence intensity between 0 and 15 were considered background. Expression of different connexins was quantified as the area under the curve (AUC) of florescence intensity histograms, and we refer to this as florescence intensity unit (FIU) in the rest of the article. AUCs and their interval estimates were calculated by using AUC analysis routine in GraphPad Prism 8.0 software (Graph Pad, La Jolla, CA, USA). Statistical significance and effect sizes as well as respective 95%CI were calculated by ANOVA followed by Tukey post hoc test in GraphPad Prism 8.0 software (Graph Pad, La Jolla, CA, USA). Level of significance was set at p = 0.05. Expression of connexins during embryonic development was analyzed by finding peaks or nadirs in time series using the single sample t test.

## Results

During early human development, the ureteric bud induces metanephric cup mesenchymal cells to gradually differentiate into renal vesicle epithelial cells, with centrally positioned lumen. The described process is known as mesenchymal-to epithelial transition^1^. During further development renal vesicle transforms into S-shaped body, which gives rise to immature glomeruli and different parts of nephron tubules.

### Co-expression of Cx43 and Cx45 in the developing and postnatal kidney tissue

**Figure 1:**
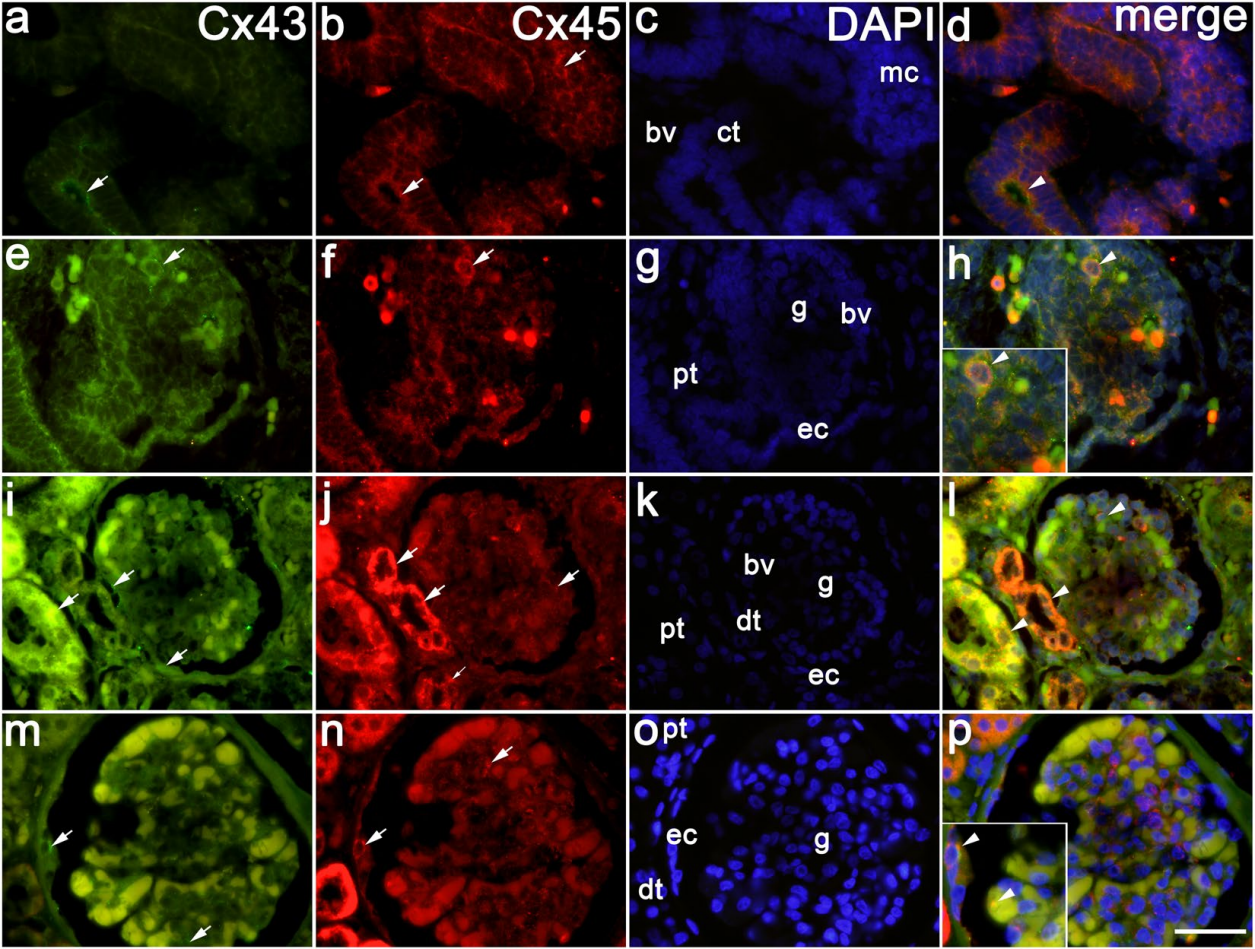
Co-expression of Cx43 and Cx45 in the developing and post-natal human kidneys. (**a**–**d**) Kidneys of 8^th^ week human embryo; (**e**–**h**) Kidneys of the 10^th^ week human foetus*;* (**i**–**l**) Kidneys of 38^th^ week human foetus; (**m**–**p**) Postnatal kidney tissue (1,5 years). expression of Cx43and Cx45 (arrows), metanephric cup (mc), collecting tubules (ct), blood vessels (bv), immature glomeruli (g), parietal epithelial cells (ec), proximal convoluted (pt) and distal convoluted tubules (dt). DAPI nuclear staining (**c**,**g**,**k**,**o**). Merged microphotographs show co-expression of Cx45/Cx43 (arrowheads) in the same cells but in different cellular compartments (**d**,**h**,**l**,**p**). Inset in (**h**) shows Cx43/Cx45 co-localization in mesangial cell, while inset in (**p**) shows their co-localization in podocytes. Double immunofluorescence staining to Cx43, Cx45 and DAPI, x100.

Double immunofluorescence method reveals co-expression of Cx45/Cx43 in the metanephric cup and collecting tubules at the earliest stages of kidney development (Fig. 1a–d). In immature glomeruli, Cx43 characterizes endothelial cells of vascular wall, while Cx45 is positioned in more peripheral part of the vascular wall (VSMC), thus Cx43/Cx45 expression mostly does not overlap. Cx45 is strongly expressed in some glomerular cells (probably corresponding to mesangial cells) (Fig. 1e–h).

In the second half of intrauterine life, strong Cx45 expression characterizes all kidney structures, while weak Cx43 expression is observed in glomeruli, proximal and distal tubules. Cx43 expression is observed in the afferent and efferent arterioles and parietal epithelial cells. Again, Cx43/Cx45 co-expression is observed in in different cellular compartments of the same structures (Table 2, Fig. 1i–l).

In postnatal kidneys, Cx43/ Cx45 co-express in different cellular or membranous compartments of the same kidney structures including podocytes, walls of blood vessels and parietal epithelial cells, Cx43 is expressed moderately, while Cx45 strongly (Fig. 1m–p, inset Fig. 1p).

### Co-expression of different Cxs with CD31 and α –SMA in developing and postnatal kidney tissue

**Figure 2:**
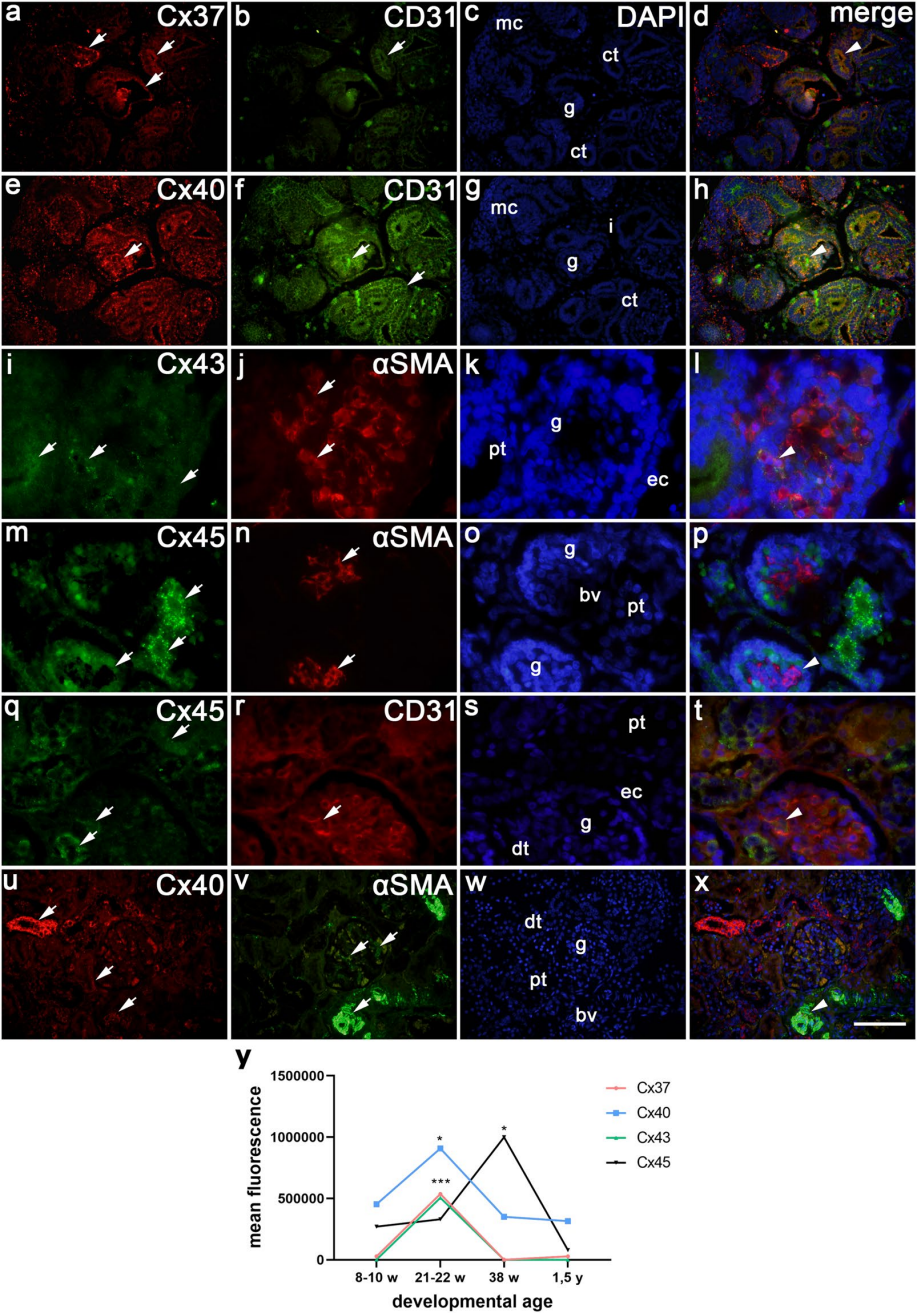
Co-expression of different Cxs (Cx43, Cx45, Cx40, Cx37) with CD31 and α –SMA in developing and post-natal human kidneys. (**a**–**d**,**e**–**h**) Kidneys of the 8^th^ week human embryo; (**i**–**l**) Kidneys of the 10^th^ week human fetus; (**m**–**p**) Kidneys of the 22^nd^ week human fetus; (**q**–**t**) Kidneys of the 38^th^ week human fetus; (**u**–**x**) Kidneys of 1,5 years old child. Cx37 and Cx40 expression (arrows), and CD31 and α –SMA expression (arrows), metanephric cup (mc), collecting tubules (ct), immature glomeruli (g), blood vessels (bv), interstitium (i), proximal convoluted (pt) and distal convoluted tubules (dt). DAPI nuclear staining (**c**,**g**,**k**,**o**,**s**,**w**). Merged microphotographs of different Cxs with CD31 and α –SMA show their co-expression (arrowheads) in the walls of blood vessels (**d**,**h**,**l**,**p**,**t**,**x**). Double immunofluorescence staining of DAPI nuclear stain with Cx37/CD31 (**a**–**d**) and with Cx40/CD31 (**e**–**h**) x20, and DAPI with Cx45/CD31 (**q**–**t**) x100. Double immunofluorescence staining of DAPI with Cx43/α –SMA (**i**–**l**), and Cx45/α –SMA (**m**–**p**), x100, and DAPI with Cx40/ α –SMA x20. (**y**) Florescence intensity histograms of different connexins during development and in postnatal period.

Between 8^th^ and 10^th^ developmental week, human kidneys contain all described stages of nephron formation, with immature glomeruli positioned closest to the future medulla. Metanephric cup cells strongly express Cx45, moderately Cx40 and Cx37, and weakly Cx43. In immature glomeruli and collecting ducts, Cx43 is expressed weakly, Cx45 strongly, while Cx40 is expressed moderately-to-strongly in the form of dense granules. Cx37 is moderately expressed in some metanephric cup cells and immature glomeruli, but strongly in collecting tubules in the form of coarse grains (Table 2, see Fig. 2. a,e and i).

Double fluorescence of CD31 with Cx40 and Cx37 (Fig. 2a–d,e–h), and double fluorescence of α –SMA with Cx43 (Fig. 2i–l), shows their co-localization in the wall of glomerular blood vessels.

During further development (10^th^ to 38^th^ week), glomeruli became mature, while proximal convoluted tubules and distal convoluted tubules become morphologically distinguishable. During that period, Cx45 is moderately expressed in the glomeruli and distal tubules, while strongly in proximal tubules (Fig. 2m,q). Cx43 slightly increases to moderate in the distal tubules, while Cx40 display moderate-to-strong expression in all three structures. Cx37 expression is moderate in the whole kidney, while weakly in the proximal tubules (Table 2).

In the 22^nd^ and 38^th^ developmental week, moderate-to-strong co-expression of Cx45 co-expresses with strong expression of CD31 and α –SMA in the walls of blood vessels (Table 1, Fig. 2m–p,q–t).

In postnatal healthy kidneys, expression of Cx45 increases only in glomeruli, while Cx43 increases from weak to moderate in all kidney structures. While Cx37 shows no changes in its expression pattern, expression of Cx40 is postnatally mild in glomeruli, moderate in proximal tubules, but increases to strong in distal tubules, (Table 2., see Fig. 2u). Double staining of Cx40 and α –SMA shows their co-localization in intraglomerular and extraglomerular blood vessels (Fig. 2u–x)

### Co-expression of different Cxs with CD31 and α –SMA in CNF

**Figure 3:**
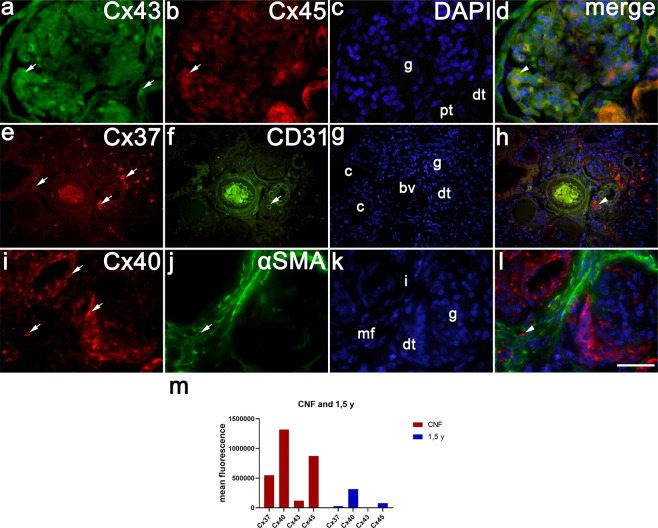
Co-expression of different Cxs with CD31 and α –SMA in CNF kidneys. (**a**–**l**) CNF post-natal kidneys (3,5 years). Cx43, Cx40 and Cx37 expression (arrows), and CD31 and α –SMA is expression (arrows), glomeruli (**g**), proximal tubules (pt) and distal tubules (dt), cysts of proximal tubules (**c**), blood vessels (bv) and interstitium (**i**). DAPI nuclear staining (**c**,**g**,**k**). Merged microphotographs of different Cxs with CD31 and α –SMA show their co-expression (arrowhead) in the wall of blood vessels and population of interstitial myofibroblasts (mf) (**d**,**h**,**l**). Double immunofluorescence staining of DAPI with Cx43 /Cx45 (**a**–**d**) x100; DAPI with Cx37/CD31 (**e**–**h**) x20, and DAPI with Cx40/α –SMA x100. (**m**) Fluorescence intensity histograms comparing healthy and CNF postnatal kidneys.

In CNF kidneys, glomeruli display different degrees of pathological changes, while some proximal tubules appear dilated thus forming small cysts. Compared to healthy postnatal kidneys, in CNF kidneys expression and distribution of different Cxs changes.: Cx45 and Cx43 expression slightly decreases in glomeruli, while Cx40 and Cx37expression increases in some distal tubules (Table 2). Cx43/Cx45 co-express in different cellular-compartments of the same kidney structures (Table 2, Fig. 3a–d). Cx37 shows irregular expression in the form of coarse granules within glomeruli, some parts of proximal and distal tubules. Double staining of Cx37 and CD31 shows their co-expression in the walls of blood vessels (Fig. 3e–h). Cx40 displays reduced expression in affected glomeruli, increased expression in distal tubules and moderate expression in interstitium, Double staining of Cx40 with α –SMA shows their co-localization in interstitial myofibroblasts (Fig. 3i–l).

### Co-expression of nephrin and synaptopodin with different Cxs in developing and CNF kidneys

Up to the 10^th^ developmental week, synaptopodin expression is absent in the kidney structures (Table 2.). In the 22^nd^ foetal week, Cx43 is mildly expressed in the glomeruli, proximal and distal tubules, and strongly in the juxtaglomerular apparatus (jga), while synaptopodin expression is moderate in the glomeruli, and very mild in proximal and distal tubules (Table 2). Nephrin expression has been previously shown to increases during human development^2^

**Figure 4:**
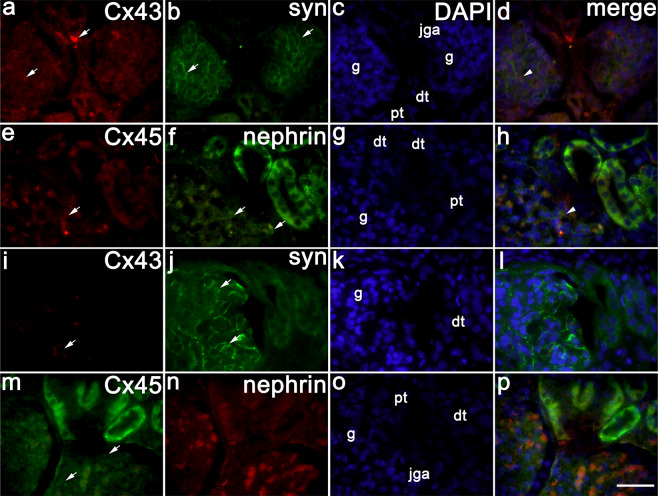
Co-expression of synaptopodin and nephrin with different Cx in developing and CNF kidneys. (**a-d**,**e–h**) Human kidneys in the 22^nd^ developmental week. (**i**–**l**,**m**–**p**) Postnatal CNF kidneys. Cx43and Cx43 expression (arrows), synaptopodin and nephrin expression (arrows), glomeruli (**g**), proximal (pt) and distal tubules (dt), juxtaglomerular region (jga). DAPI nuclear staining (**c**,**g**,**k**,**o**). Merged view of Cx43 or Cx45 with synaptopodin shows co-expression of the two markers (arrowheads) in the glomeruli (podocytes) of healthy kidneys (**d**,**h**), and absence of their co-expression in CNF kidneys (**l**,**p**). Double immunofluorescence staining of DAPI with Cx43 or Cx45 with nephrin or synaptopodin x100.

Synaptopodin only partly co-expresses with Cx43 in the glomerular cell population probably corresponding to podocytes (Fig. 4a–d), as well as does Cx45 with nephrin (Fig. 4e–h).

CNF kidneys show decreased and only partial expression of synaptopodin in the glomeruli, and very weak expression in proximal and distal tubules (Table 2; Fig. 4i–l). Cx43 and synaptopodin do not co-express in the podocytes (Fig. 4i–l), while nephrin expression in CNF glomeruli is very mild (Fig. 4m–p).

### Co-expression of different Cxs with markers of proximal tubules (DBA) and distal tubules (LTL) in developing, postnatal and CNF kidneys

**Figure 5:**
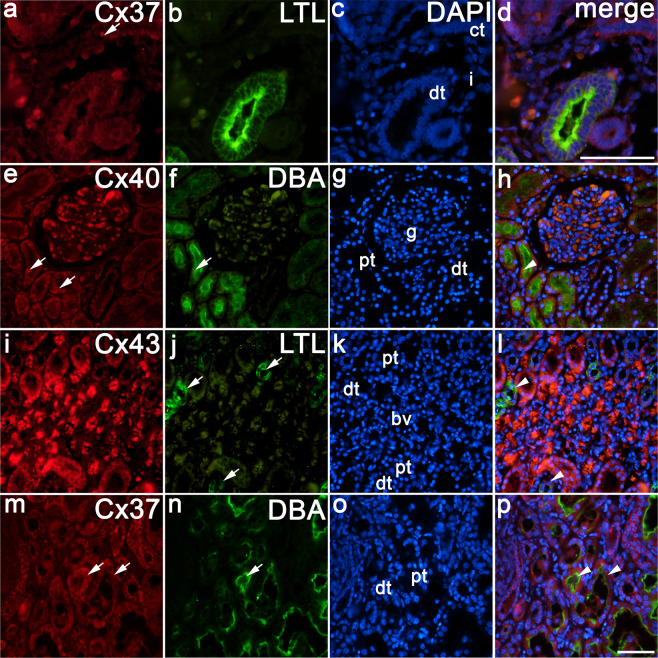
Co-expression of different Cxs with markers of proximal tubules (DBA) and distal tubules (LTL) in developing, postnatal and CNF kidneys. (**a**–**d**) Human kidneys in the 10^th^ developmental week. (**e**–**h,i**–**l)** Human postnatal kidneys (1,5 years). (**m**–**p**) Postnatal CNF kidneys (3,5 years). Cx37, Cx40 and Cx45 expression (arrows), expression of markers for proximal (DBA) and distal (LTL) tubules (arrows), proximal tubules (pt), distal tubules (dt), collecting tubules (ct), interstitium (i), glomeruli (g), blood vessels (bv). Co-expression of Cx37 with LTL is missing in 10^th^ developmental week kidneys (**a**–**d**), while co-expression of Cx40/DBA (arrowhead) (**e**–**h**) and Cx43/LTL (arrowhead) (**i**–**l**) is seen in proximal and distal tubules of postnatal kidneys. In CNF, Cx37 co-expresses with DBA (arrowhead) in dilated proximal tubules. Double immunofluorescence staining of DAPI with Cx37/LTL, Cx40/DBA, Cx43/LTL and Cx37/DBA x40.

In 8^th^ to 10^th^ developmental week, only small number of kidney tubules showed strong signal to LTL, while there was no signal to DBA at that developmental stage. Double staining of Cx37 with LDL shows absence of their co-expression in distal tubule, while positive signal to Cx37 was seen in collecting ducts (Table 2, Fig. 5a–d). In contrast to developing kidneys, postnatal kidneys showed strong reactivity to both DBA in proximal tubules and LDL in distal tubules. Double staining of Cx40 with DBA showed their co-expression in proximal tubules (Fig. 5e–h), while Cx43 co-expressed with LDL in distal tubules (Fig. 5i–l).

In CNF kidneys, dilated proximal tubules strongly reacted with DBA. Double staining of Cx37 with DBA shows their co-expression in proximal tubules (Fig. 5m–p).

By using specific markers for proximal and distal tubules, we could prove that morphological criteria for detection of different parts of nephron tubules in our study accorded with their labelling with specific markers for proximal and distal tubules.

### Electron microscopy

**Figure 6:**
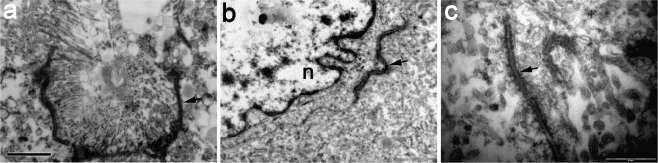
Transmission electron microscopy (TEM) of the connexins in the developing kidney tissue and CNF kidneys. (**a**) Connexins (arrows) are observed between the tubular cells in the 6^th^ developmental week. (**b**) Connexins (arow) interconnect glomerular cells in the kidney tissue of the 21^st^ week human embryo. (**c**) Connexins (arrows) between the tubular cells in the CNF kidneys.

In the 6^th^ developmental week, abundant gap junctions interconnect epithelial tubular cells (Fig. 6a). During progression of development gap junctions are found in different parts of kidney tissue, including glomerular cells (Fig. 6b). In the CNF kidneys, besides typical depletion of podocyte pedicles, we observed connexins in the whole kidney tissue, but most abundantly between the tubular cells (Fig. 6c).

### Quantification and semi-quantification of Cxs expression during development and postnatal period

*Semi-quantification* of Cxs expression in different parts of nephron is shown in Table 2.

*Analysis of the mean fluorescence signal* of different Cxs revealed significant difference of maximal signal (peak signalling) between Cxs: Cx37, Cx43 and Cx40 displayed very similar course of fluorescent intensity signalling, being strongest in the 21^st^ developmental week, but overall intensity of Cx40 was stronger (p = 0.0003, p = 0.02, p < 0.0001, respectively). The strongest fluorescence signalling characterized Cx45, with peak in the 38^th^ developmental week (p = 0.03). In postnatal period, intensity of florescence signal dropped, except for Cx40 which remained high (Fig. 2y). All Cxs signals are much stronger in CNF kidneys compared to healthy postnatal kidneys (Fig. 3m).

## Discussion

Connexins are transmembrane proteins that form gap junctions, which in the kidney tissue primarily contribute to renal haemodynamics, as they mostly localize in the walls of blood vessels. However, they also seem to have essential role in tubular epithelial and glomerular function^42^, in prenatal development, morphogenesis and tissue differentiation. By now, numerous experimental studies resulted in controversial data about spatio-temporal expression of specific Cx sub-types in the kidney tissue.

In the present study, strong expression of Cx45 displayed decreasing course in glomeruli of developing kidneys, while its strong expression in proximal tubules continued into postnatal period. In contrast, during development of mouse kidneys, Cx45 expression paralleled appearance of renin producing cells, but postnatally disappeared^38^. Compared to healthy postnatal kidneys, CNF kidneys showed disorganized distribution of Cx45 in proximal tubules, what might be associated with faulty signalling. CNF has been previously shown to be histologically characterized by tubular primary cilia dysregulation and appearance of proximal tubules cysts^43^. We also found both Cx45 and Cx40 expression in glomerular mesangial and granular cells, thus confirming possibility of appearance of heterotopic channels between different Cxs, which has been shown in experimental animals^19,38,44^. However, use of different techniques in studies analysing Cxs expression led to conflicting conclusions. Thus, Cx45 was detected in mouse distal tubules by immunohistochemistry^23^ but its mRNA failed to be confirmed^32^.

In our study, different kidney structures showed weak Cx43 expression, which during development gradually increased to mild only in distal tubules. While Cx43 expression increased in glomeruli and proximal tubules of postnatal healthy kidneys, it became irregular and strong in distal tubules of CNF kidneys. In contrast to our results, previous study on human foetal kidneys missed to show early Cx43 expression in the metanephric cup cells^34^, what might be explained by higher sensitivity of immunofluorescence antibodies used in the present study. Discrepancies in the reported expression of CX43 and Cx45 might also reflect interspecies differences in Cxs expression^20^. In comparison to Cx45, Cx43 expression was confined only to apical cytoplasmic compartment of tubular nephron cells, while Cx43 / Cx45 co-expression characterized different cellular compartments in mesangial cells, podocytes, and parietal epithelial cells. Our study also showed that both Cx43 and Cx45 co-localized with markers for podocyte pedicles, thus pointing to importance of those two Cxs subtypes in podocyte signalling. Dedifferentiation of human podocytes in CNF kidneys has been previously shown to be associated with changes in cytoskeletal arrangement, presence of primary cilia and re-expression of nestin^1,2^. Additionally, interactions between connexins were suggested to be crucial in regulation of EMT of developing kidney cells^45^, which in CNF seems to be disturbed.

Studies on animals showed that Cx43 might be primarily involved in vasomotion, but also in gene transcription, ATP and vesicle release, cytoskeletal dynamics and cell stress^11^. In CKD, deletion of Cx43 caused anti-inflammatory effect and reduced interstitial fibrosis, while similar to our study - its disruption led to depletion of podocyte pedicles^46^. In contrast, Cx43 upregulation promoted pro-inflammatory environment^21^ thus suggesting that alterations in expression of Cxs might be associated with development of CKD^37^.

During development, moderate-to-strong expression of Cx37 in human kidney structures continued into postnatal period. Similar to our study, previous animal studies have also shown expression of Cx37 primarily in distal nephron^20^. Importance of early expression of Cx37 and Cx43 was implicated in kidney development, while their elevation was referred in neonatal unilateral ureteral obstruction^32^. We also noticed overall elevation of Cx40 and Cx37 expression in CNF kidneys, particularly in distal tubules.

Moderate-to strong expression of Cx40 during human kidney development decreased in glomeruli and increased in distal tubules in postnatal period. Opposite to our study, Cx40 expression in rats was observed in intra and extraglomerular mesangial cells and non-glomerular endothelial cells^29^.

While use of different techniques in analysing Cxs expression in kidney tissue often led to conflicting results, application of immunohistochemical method in kidney tissues of different animal species and humans revealed a certain degree of conservation in their spatio-temporal expression.

In conclusion, in developing human kidneys co-expression of different Cxs with markers for endothelial cells (CD31) and VSMC (α –SMA) indicated importance of communications between those two cell populations for vascular wall integrity. Overall signalling intensity of Cx37, Cx43 and Cx40 had its peak in the 22^nd^ developmental week and accompanied a process of nephrogenesis, while peak of Cx45 expression in the 38^th^ week corresponded to period of functional nephron maturation^47,48^. Based on the Cxs spatiotemporal expression in glomerular and tubular human kidney cells, we suggested importance of Cx45 in differentiation of proximal tubules, and Cx43, Cx37 and Cx40 in differentiation of distal tubules. Additionally, fine cytoplasmic balance between Cx43 and Cx45 co-expression might be crucial for differentiation of glomerular cell population, including podocytes, mesangial and parietal epithelial cells. The observed changes in Cxs expression characterized all structures in developing and CNF kidneys, thus pointing to Cxs as possible target in treatment of kidney diseases and repair processes^29^. Alterations in gap junction activity have been shown to cause structural and functional damage in several kidney diseases^21,29,37^. We also showed increased number of myfibroblasts co-localizing with Cx40 in the interstitium of CNF kidneys, thus implying possible involvement of Cx40 in regeneration processes^49^. Namely, expansion of myofibroblast population, which acquired some functional and structural characteristics of smooth muscle cells^50^ lead to extensive deposition of interstitial extracellular matrix, which is the key characteristic of renal fibrosis in CKD. In addition, some dedifferentiated renal fibroblasts re-expressed mesenchymal markers, what might be considered as a sign of EMT^6^. Therefore, treatment that would re-establish mature fibroblast phenotype could be another option for treating renal fibrosis^6,51^.
